# Periorbital lesions in severely burned patients

**Published:** 2019

**Authors:** Andreea Grosu-Bularda, Mihaela-Cristina Andrei, Anca Daniela Mladin, Maria Ionescu Sanda, Maria-Magdalena Dringa, Dragos Constantin Lunca, Ioan Lascar, Razvan Nicolae Teodoreanu

**Affiliations:** * Clinic of Plastic Surgery, Aesthetic and Reconstructive Microsurgery, Clinical Emergency Hospital Bucharest, Romania; ** Ophthalmology Department, Clinical Emergency Hospital Bucharest, Romania; *** Ophthalmology Clinic, “Dr. Carol Davila” Central Military University Emergency Hospital, Bucharest, Romania; **** ”Carol Davila” University of Medicine and Pharmacy, Bucharest, Romania

**Keywords:** periorbital burns, negative prognostic factors, corneal burns, exposure keratopathy

## Abstract

Purpose: This study aimed to characterize the injuries involving periorbital region in our severely burned patients.

Method: A 2 years retrospective study was conducted with a total of 210 severe burns admissions. Periorbital burn injuries (all produced in association with facial injuries) were encountered in 126 patients, representing the study group that was further analyzed for multiple parameters: demographics, mechanism of injury, TBSA (total body surface area), burn depth, inhalation injury, need for intubation and mechanical ventilation. The presence and severity of ocular injuries were also evaluated.

Results: Analyzing our study group (n=126), we observed the presence of multiple negative prognosis factors: elderly patients, extensive burns, deep burns affecting functional areas, unfavorable mechanism (electric, chemical or explosions), inhalation injuries, need for intubation and mechanical ventilation, leading to severe morbidity and high mortality level.

Ocular injuries were encountered in 37 patients (30 primary and 7 secondary lesions). The predominance of primary ocular lesions is explained trough high severity burns encountered in our patients with high mortality and lack of long-term clinical observations.

Conclusion: The clinical outcome for periorbital burn injuries depends on patient characteristics, etiology, burn extension and depth, associated lesions, infectious risk and the quality of the treatment applied. Presence of ocular injuries in various severity degrees impose an adequate evaluation and specialized treatment, being associated with important morbidity. In severely burned patients, it is mandatory to apply preventive measures to avoid ocular complications. If exposure keratopathy is detected, prompt ophthalmologic treatment is essential to avoid functional impairment including loss of vision.

**Abbreviations:** TBSA = total body surface area, MSOF = multisystem organ failure, OCS = orbital compartment syndrome, AION = anterior ischemic optic neuropathy

## Introduction

Severe burn injuries represent a major challenge to the entire healthcare system, with a worldwide estimation of 300 000 deaths/ year determined by burns, especially in poor countries. According to World Health Organization available reports, in 2004, worldwide, almost 11 million people required medical treatment for burn injuries [**1**,**2**]. In USA, each year, around 500 000 burned patients need medical treatment, approximately 40 000 of them require hospital admission and 3400 deaths caused by burns are registered annually [**3**]. 

For their survivals, burn injuries are a major cause of morbidity, necessitating prolonged hospitalization with subsequent complications, with further disabilities and disfigurement, resulting in stigmatization and social rejection [**1**,**2**]. 

In patients with extensive burns, usually the face is affected. As an essential functional and aesthetic region, facial burn lesions lead to important physical and psychosocial morbidity. Periorbital and ocular injuries are reported in literature with a proportion of 20% in facial thermal burns [**4**]. 

The severity of the periorbital burn injury and future prognostic depends on patient characteristics, mechanism, duration of exposure to traumatic agent, isolated lesion or associated in context of extensive burns, extent of the tissue damage, infectious risk and the quality of the treatment applied [**4**-**12**]. 

Periocular area lesions can be determined through various mechanisms including thermal, chemical, and electrical or radiation burn injuries, detailed in **Table 1** [**12**]. 

**Table 1:** Mechanisms of burns

Mechanism	Agents
Thermal burns	Flames
	Flash/ explosion
	Hot liquid or steam
	Contact with hot objects
Electrical burns	Electrical sources: low or high voltage injuries
	Lightening
Chemical burns	Alkali
	Acids
	Peroxides
	Detergents/ solvents
Radiation injuries	Ultraviolet rays
	Ionizing radiation
	Lasers

**Fig. 1:**
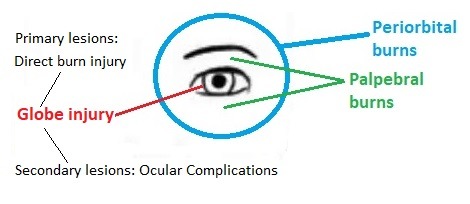
Periorbital region burns extension

Extension and severity of burns involving periorbital region are variable (**Fig. 1**), with different clinical outcome [**5**,**12**].

**Fig. 2:**
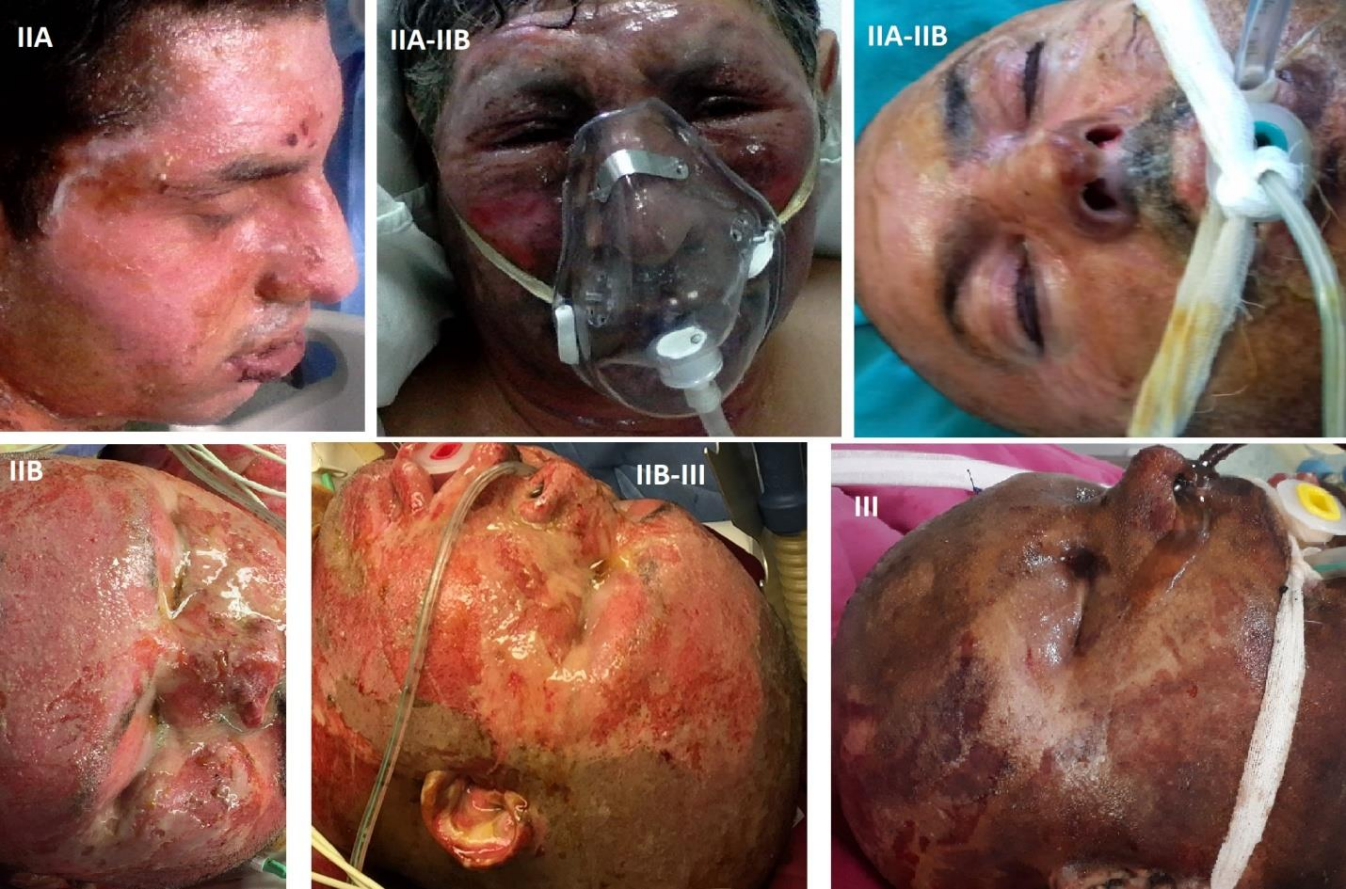
Clinical aspects of burn lesions in our patients

Depending on how deep the skin is affected, burns are classified as first-, second-(IIA+IIB) and third degree, as we can see in **Table 2** [**13**,**14**]. **Fig. 2** illustrates various depth burn lesions encountered in our patients

**Table 2:** Depth of burns

	Degree of burns			
	I	IIA	IIB	III
Depth	Superficial	Partial thickness	Partial deep thickness	Full thickness
Clinical aspect	Erythematic, pain	Blisters with serous content, painful	Hemorrhagic blisters, red and white spots, less painful	Burn eschar, painless
Skin layers involved	Epidermis	Epidermis + reticular dermis	More than reticular dermis, not the entire dermis	Dermis is completely burned
Healing time	<1 week	2-3 weeks	>3 weeks	No spontaneous healing; impose surgical treatment

Two classification systems are currently used for corneal injuries: Roper Hall and Dua classifications [**7**,**15**]. **Table 3** presents the elements included in the Roper Hall classification and clinical prognostic depending of the extent of the lesions.

**Table 3:** The Roper Hall Classification

Grade	Corneal injury	Limbal Ischemia	Prognostic
I	Epithelial damage	NO	Good
II	Haze, iris is visible	<1/ 3	Good
III	Complete epithelial loss, iris details are obscured	Between 1/ 3-1/ 2	Reserved
IV	Opaque cornea with iris and pupil covered	>1/ 2	Poor

The Dua Classification has six grades, evaluates limbal injury in clock hours and the percentage of affected bulbar conjunctiva, therefore Roper Hall fourth grade injuries are classified by Dua in another three grading categories, from four to six, depending of the extent of the injuries [**7**].

In patients with severe palpebral or adjacent facial burns without or with minimal initial injury of the eyeball, secondary important lesions may occur through corneal exposure and possible association of infections [**5**,**12**].

**Fig. 3:**
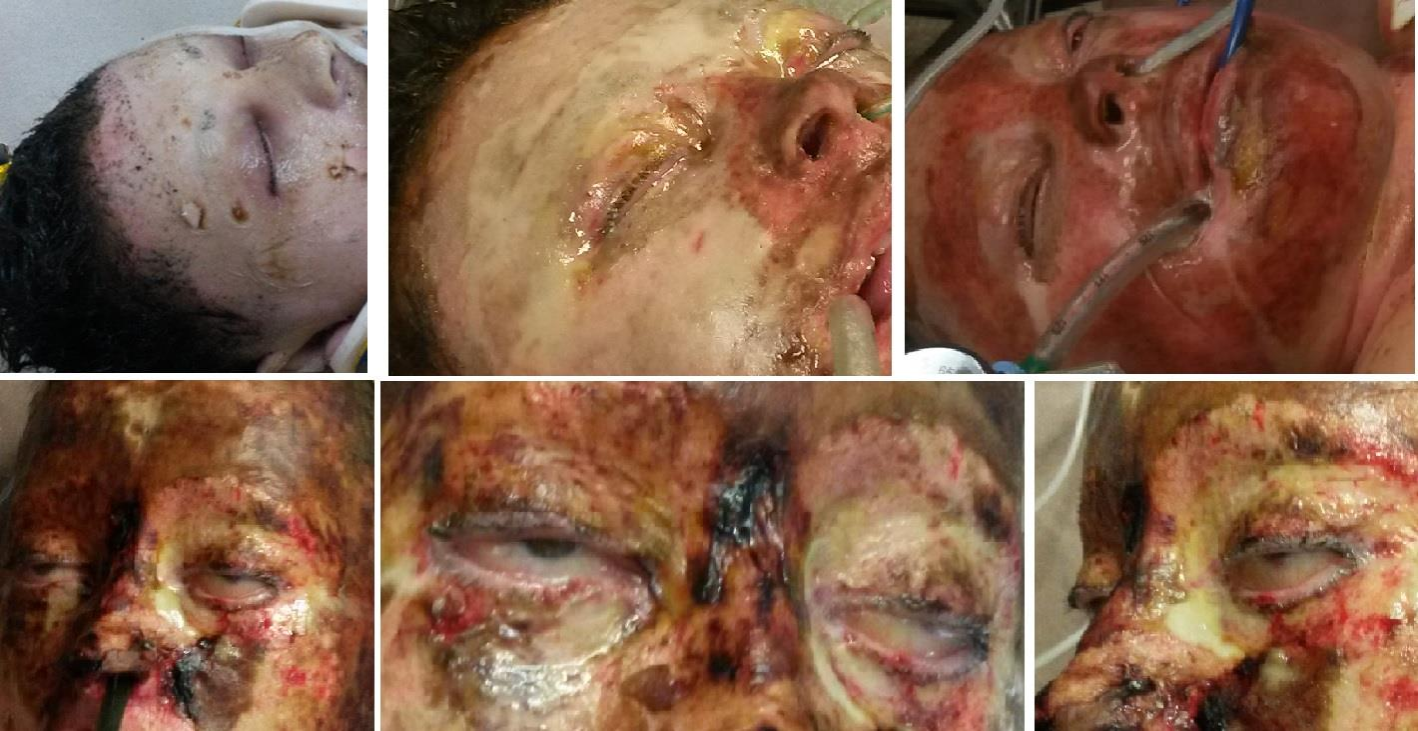
Ocular complication in a case of a deep, extensive burn in a 41-year-old female patient: periorbital deep burns with palpebral retraction and corneal exposure

**Fig. 4:**
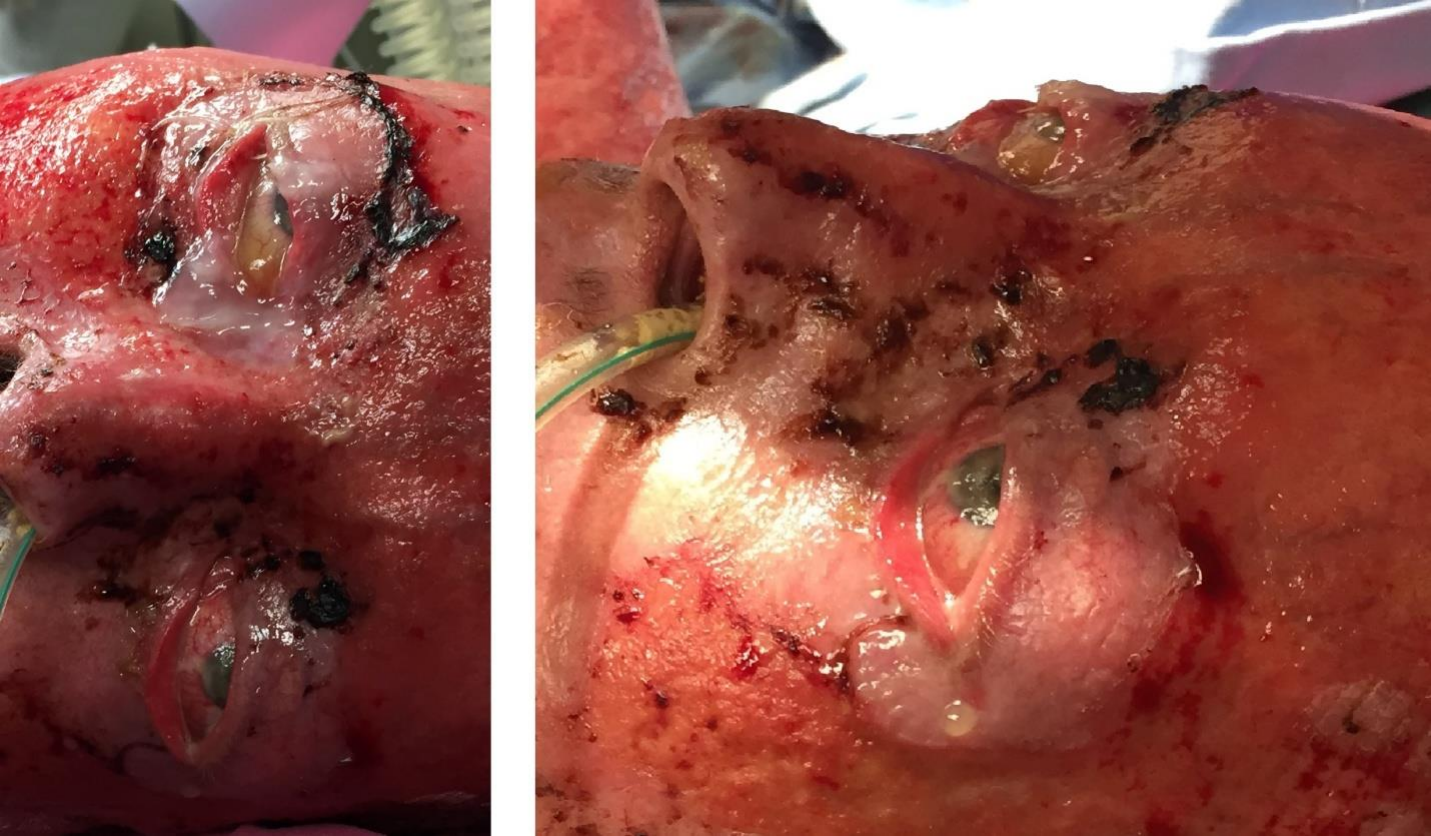
Ocular complication of bilateral cicatricial ectropion and corneal exposure in a case of a deep, extensive burn in a 46-year-old male patient

Through **Fig. 3** and **4** we present the ocular complications developed in two of our patients with deep, extensive burns of the periorbital region, noting the particularities of these types of complications. 

Appropriate prompt therapy of periorbital injuries is an important determinant on the burned patient clinical outcome [**12**].

In the emergency phase after the severe burn occurrence, immediate intervention is mandatory, with stabilization of vital functions, initiation of fluid resuscitation and transport of patient to a specialized critical care burn unit to receive the adequate treatment. Detailed clinical and paraclinical examination is performed by a multidisciplinary team: the emergency physician, plastic surgeon, anesthesiologist, general surgeon, neurosurgeon, orthopedic surgeon, ophthalmologist, ENT physician and cardiologist; laboratory tests and imagistic evaluation are conducted. An important aspect is to obtain an accurate microbiological screening on admission, from multiple body sites involving burned and unaffected areas. The extent and depth of the burn lesions are established by an experienced burn surgeon, associated lesions (especially inhalation injuries) are carefully investigated and also patient history and comorbidities are noted.

**Fig. 5:**
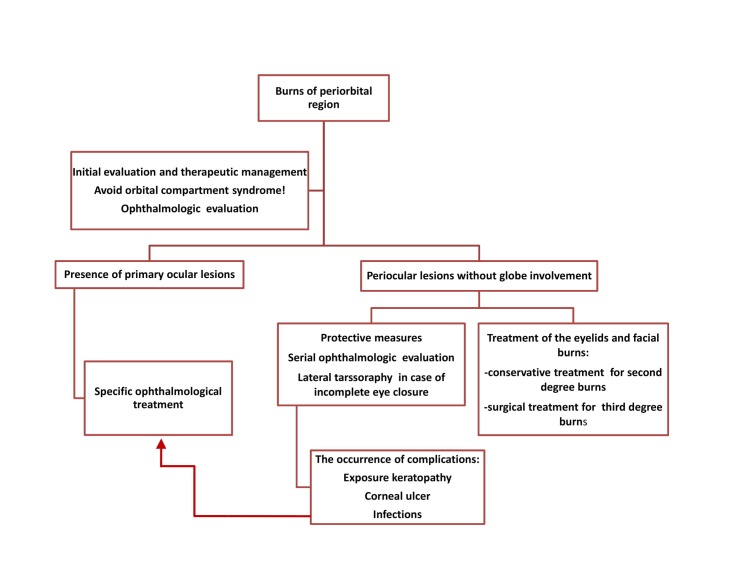
The therapeutic principles in periorbital burn injuries [**4**,**5**,**12**]

**Fig. 6:**
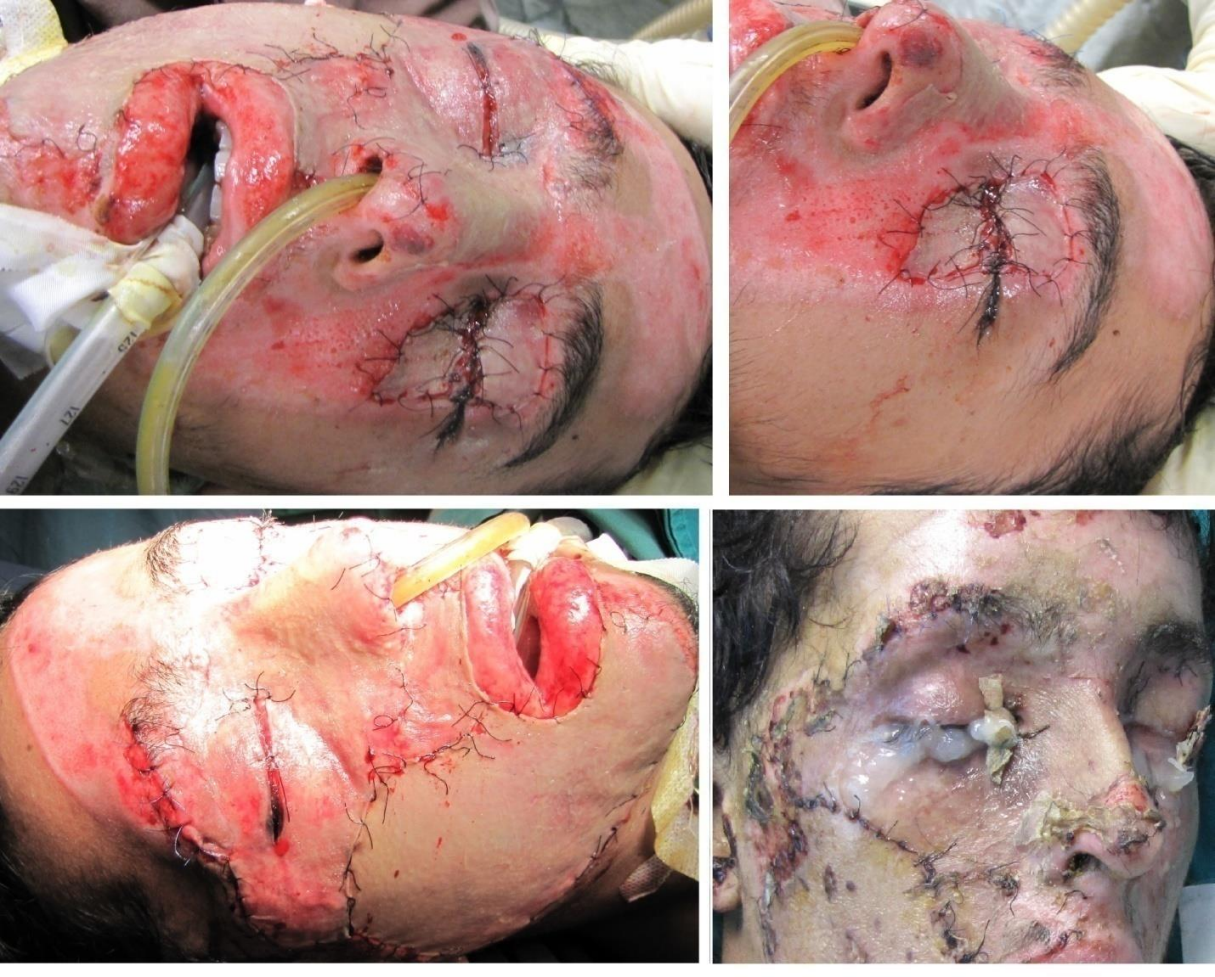
Facial and palpebral full sheet skin grafting in a 20 years old patient presenting extensive deep burns

The following measures are required in emergency phase for periorbital burns: washing of the burn wounds and removing the foreign bodies, disinfection of the area, opening of the eyelids and abundant irrigation of the eye with saline solution or Ringer lactate (especially in case of chemical burns), extraction of foreign particles. During the following days, the patient is evaluated in dynamics by the ophthalmologist and therapeutic measures are decided depending of the extent of the ocular lesions: protective measures to keep the eyeball covered, topical application of artificial tears, topical antibiotics when necessary, temporary tarsorrhaphy addressing lagophthalmos when eyelids are severely burned, use of conjunctival flaps for globe coverage, deep, full thickness burn wounds excision and skin grafting to prevent further complications. Severe destruction of the ocular globe is rare, but may impose enucleation. The therapeutic principles regarding periorbital burns are synthesized in **Fig. 5** [**4**,**5**,**12**]. **Fig. 6** presents facial and palpebral full sheet skin grafting in a young male patient with extensive deep burns. 

## Methods

A two years (01.05.2016-01.05.2018) retrospective study was conducted on the patients admitted in the Critical Care Burn Unit of Clinical Emergency Hospital Bucharest. Data were obtained from medical records and the hospital’s eHealth program (Hipocrate), then were centralized and analyzed using Microsoft Excel. From all the hospitalized patients in our burn unit, we noted the ones with facial burns and determined the group with involvement of the periorbital region. 

Multiple parameters were evaluated for each patient: demographics, mechanism of injury, TBSA, burn depth, presence of third degree burns, presence of inhalation injury, need for intubation and mechanical ventilation. A thorough analysis was performed for patients with burns of periorbital region, noting previous parameters as well as the presence and severity of ocular injuries (including primary burn lesions and ocular complications developed during hospitalization). Corneal injuries were classified according to Roper-Hall international classification (**Table 3**). Therapeutic management was also recorded. The study adhered to the Declaration of Helsinki and to our institutional and national ethical regulation. 

## Results

**Fig. 7:**
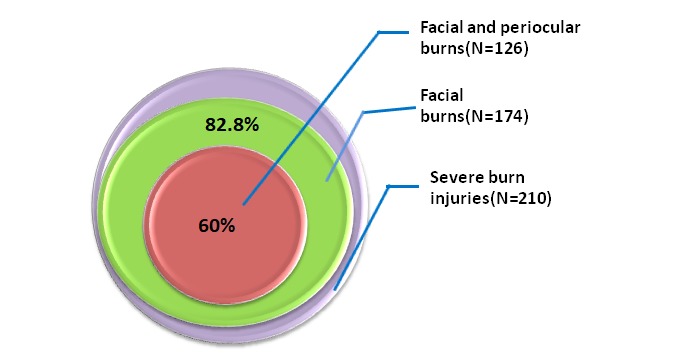
Periorbital and facial burns diagram

**Fig. 8:**
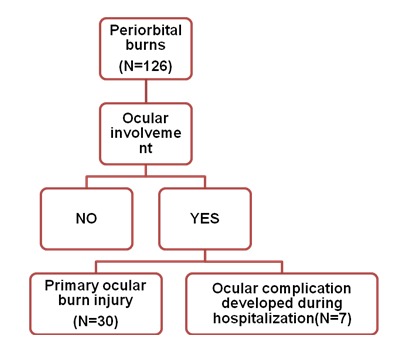
Ocular involvement in periorbital burns

During the analyzed period, 210 patients were addmited in our Critical Care Burn Unit. We analyzed those critical patients in order to determine injuries of periorbital area involvement. In our cases, all periorbital injuries (encountered in 126 patients) were produced in association with facial injuries (the distribution is seen in **Fig. 7** and **8**). The study group with periorbital burn injuries was further analyzed for multiple parameters as seen bellow. 

**Graph 1:**
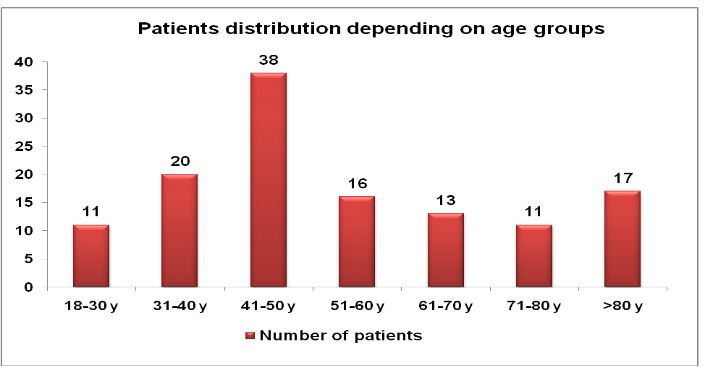
Age distribution diagram

The provenience of the patients was from rural areas in 55.5% of the cases and urban areas in 44.5% of the cases. Gender ratio was approximately 2:1, with male patients predominance: 83 cases (66%) male patients and 43(34%) female patients. The age of patients ranged from 19 to 96 years (**Graph 1**), with an average of 53.6 years (the average age of patients in all the 210 critical care burn unit admissions was similar - 55.3 years). 

**Graph 2:**
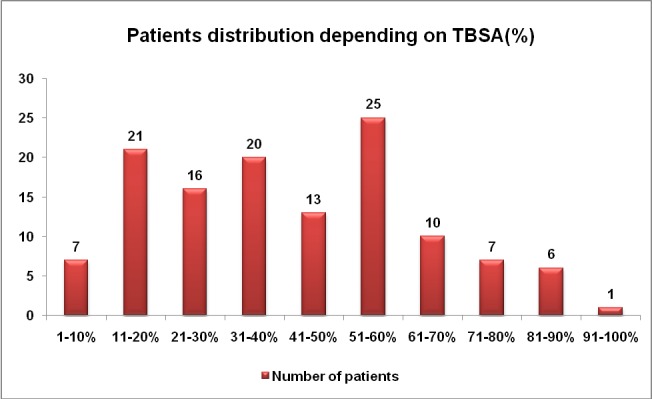
TBSA distribution of patients with periorbital lesions

**Graph 2** number shows extension of the burns in patients with periorbital lesions; the average TBSA (total body surface area) was 44% and the median TBSA was 40%. Extensive burns exceeding 40% TBSA were encountered in 49.2 % of the patients. 

**Graph 3:**
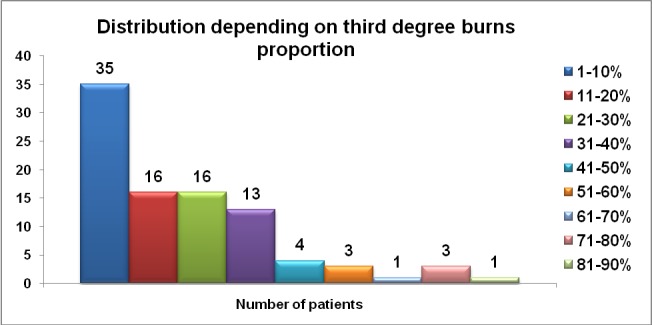
Full thickness burns distribution

For the patients with periorbital burns, 92 (73%) of them presented third degree burns on their body (**Graph 3**).

**Graph 4:**
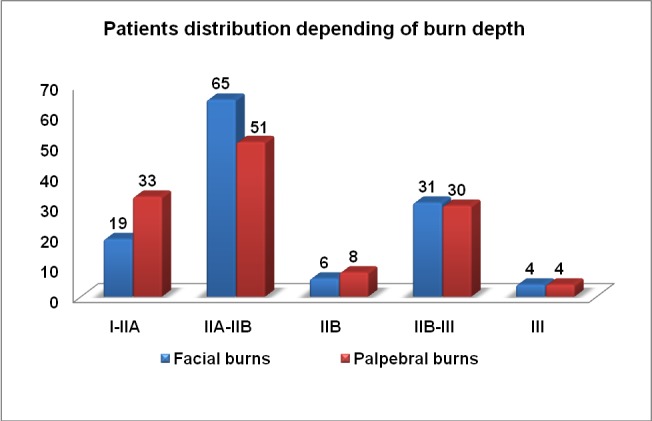
Distribution of burn depth in the face and palpebral region

Deep lesions were also observed in the face and particularly in the periorbital region; the distribution of burn depth in the face and palpebral region is illustrated in **Graph 4**. 

**Fig. 9:**
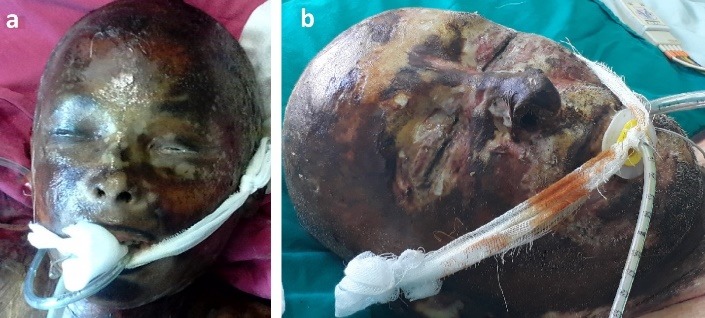
Extensive facial full thickness burns in two cases: (a. 67-year-old woman and b. 84-year-old man)

Four patients had extensive full thickness burns involving the entire face, with palpebral and ocular lesions, with very poor prognosis (two of them presented in **Fig. 9**). All these four patients died. 

**Graph 5:**
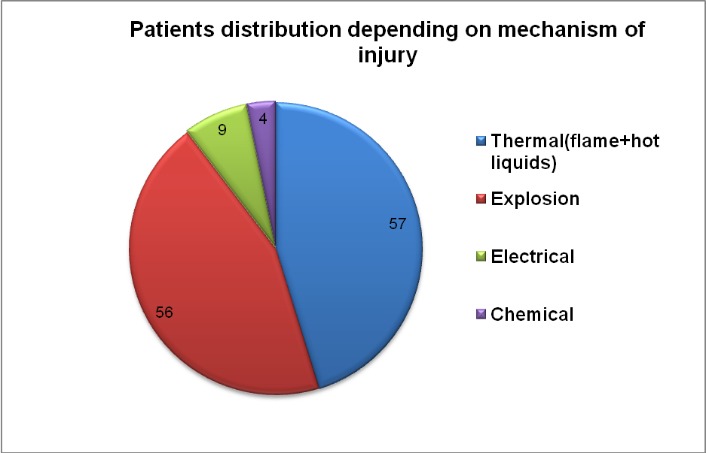
Mechanism of injury

Regarding the mechanism of periorbital burns, we encountered a number of 56 explosions (44.4% of the patients), 7% electrocutions, and 3% chemical burns, as seen in **Graph 5**, the largest proportion in our group being thermal injuries. 

In 13 patients (10.3%), the lesions were work-related burns. As severity factor, 100 patients (79.3%) associated inhalation injuries. From the 9 patients with electric injuries, 7 were represented by high voltage electrocutions, 1 case - electric flame injury and one patient had a low voltage injury. The chemical injuries were produced by sulfuric acid in 3 cases (two cases from industrial source-work related injury and, in one case, battery acid was the cause) and by Sodium hydroxide (caustic soda) in one patient. 

**Graph 6:**
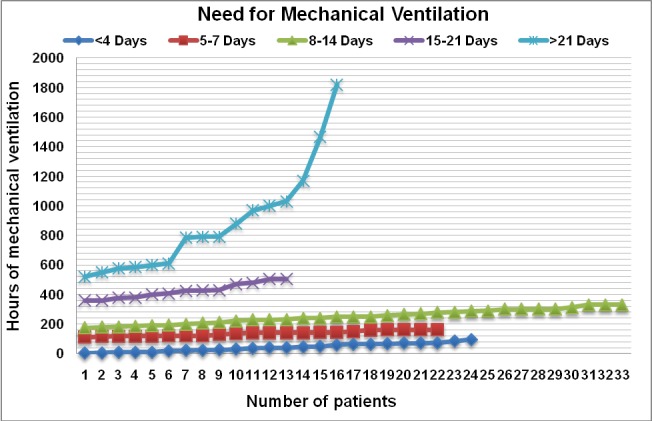
Time of mechanical ventilation required

In our group of patients with periorbital burns, 108 of them (85.6%) required intubation and mechanical ventilation (**Graph 6**), with an average of 296 hours (12.3 days) of mechanical ventilation, the median being 208 hours (8.6 days). 

The mortality rate was very high in the study group: from 126 patients being 84 (66,7%) deceased cases.

**Ocular injuries**

**Patients with primary ocular burn injuries**

**Graph 7:**
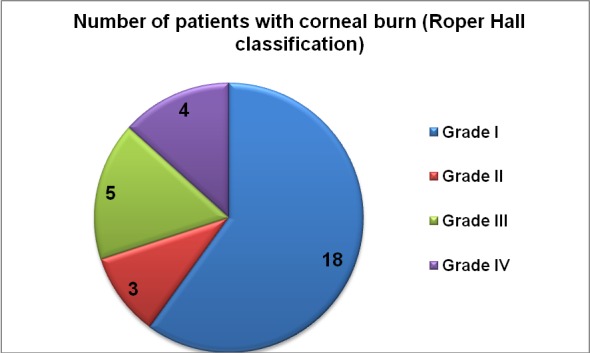
Roper Hall classification of our subgroup of patients with corneal burns

Corneal injuries were encountered in 30 patients, representing 23.8% of periorbital burn injuries. From the patients with corneal injuries 23 were males and 7 were females (sex ratio 3:1), the average age was 50.1 years, the average TBSA was 43.9%. We evaluated those injuries according to Roper Hall classification as highlighted in **Graph 7**.

In the subgroup presenting corneal injuries, the mortality was even higher, with 25 of 30 (83.3%) deaths. All the 12 patients with corneal injuries grade II-IV died, having severe burn injuries.

**Graph 8:**
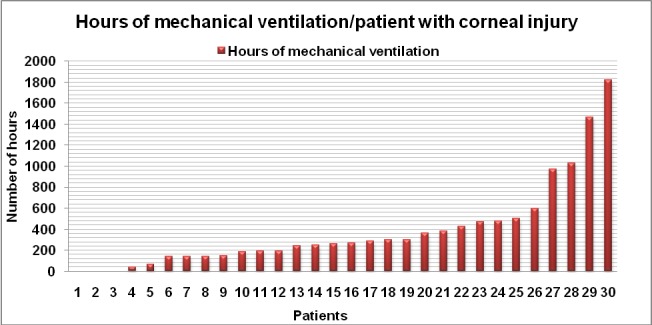
Hours of mechanical ventilation required in patients with corneal injury

In the group of patients with corneal injury, 27 of them (90%) required intubation and mechanical ventilation (**Graph 8**), with an average of 432 hours (18 days) of mechanical ventilation, the median being 288 hours (12 days). Surgical treatment involving excision of full thickness eyelid burns and full thickness skin grafting was performed in 2 patients (in one of them bilateral lateral tarsorrhaphy was performed), both of them dying because of serious burns complications. 

**Fig. 10:**
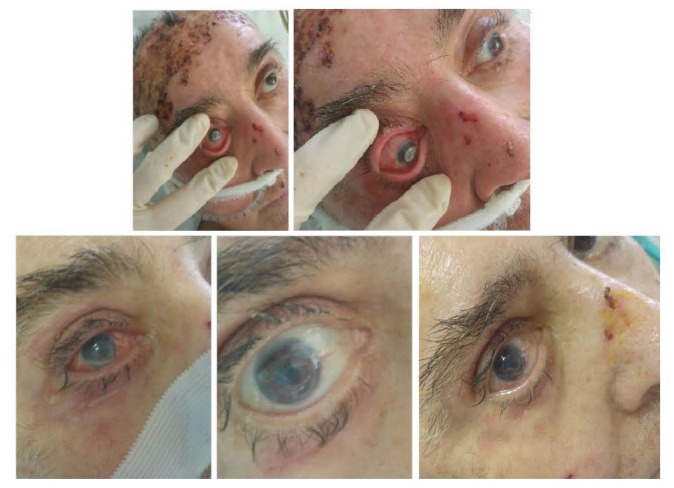
Corneal ulcer with hipopion complication and bacterial infection with Pseudomonas aeruginosa, aspect in succesive evaluations

We present the case of a 60-year-old male (**Fig. 10**), victim of an explosion injury, with 25% TBSA burns, grade IIA-IIB-III (10% third degree lesions), associating inhalation injuries, being the patient with the longest intubation period from this group - 1820 hours/ 76 days). Periorbital lesions were IIa-IIB grades, with corneal involvement evaluated as Roper-Hall grade 1. In evolution, despite protective mesures and topical ocular treatment, the patient developed corneal ulcer with hypopion at eight weeks from admission. Microbiological testing showed the presence of bacterial infection with multiresistent Pseudomonas aeruginosa, having sensibility only to Colistin and Amikacin. The evolution was favorable under topical ocular antibiotic treatment with Colistin. The patient died due to systemic burn complications after 79 days post-burn injury.

**Ocular complications developed during hospitalization**

Seven patients (5 men and 2 women, with age between 41 and 59 years - an average age of 47 years) developed secondary ocular complications during their long-term hospitalization: they had between 21 and 202 hospitalization days, with an average of 72 days and a median of 51 days. The mechanism of injury was thermal burns, in four of the patients involving explosion. 

**Graph 9:**
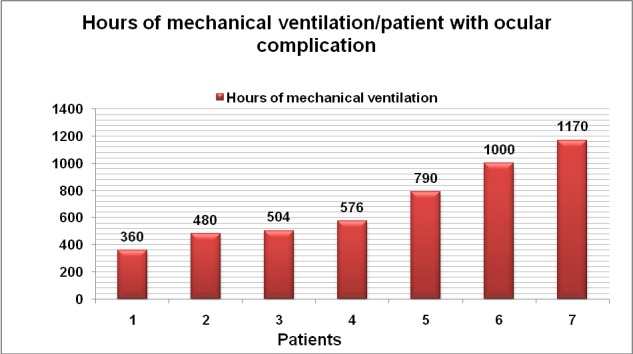
Hours of mechanical ventilation required in patients with ocular complications

All those seven patients required intubation and mechanical ventilation (**Graph 9**), the average being 697.1 hours (29 days) of mechanical ventilation with a median of 576 hours (24 days); six of them presented inhalation injuries, 3 of them survived and 4 died. 

One patient developed moderate lagophthalmos with exposure keratitis at two weeks post-injury than in one month post-burn injury, an inferior corneal ulcer with superficial corneal abscess developed in left eye; microbiological tests revealed the presence of Acinetobacter baumannii and antibiotic treatment was conducted according to serial antibiograms; blepharorrhaphy on the left eye was performed at 2 months post-burn injury with initial favorable result, but the long term observation of evolution was not possible, due to patient death 2 weeks after the blepharorrhaphy. Other 3 patients developed incipient forms of keratopathies due to palpebral retraction and corneal exposure, topical treatment was conducted, one patient survived with good evolution and no further ocular complications and hospital discharged after 47 days and the other two patients died. 

**Fig. 11:**
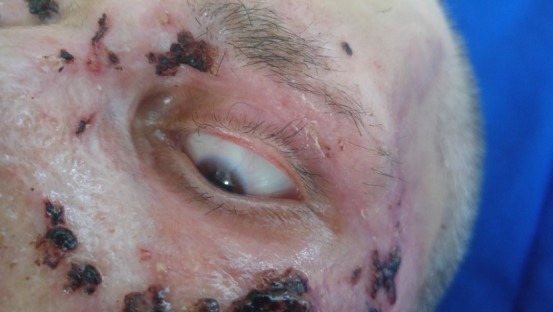
Cicatricial ectropion on the left eye and lagophthalmos

**Fig. 12:**
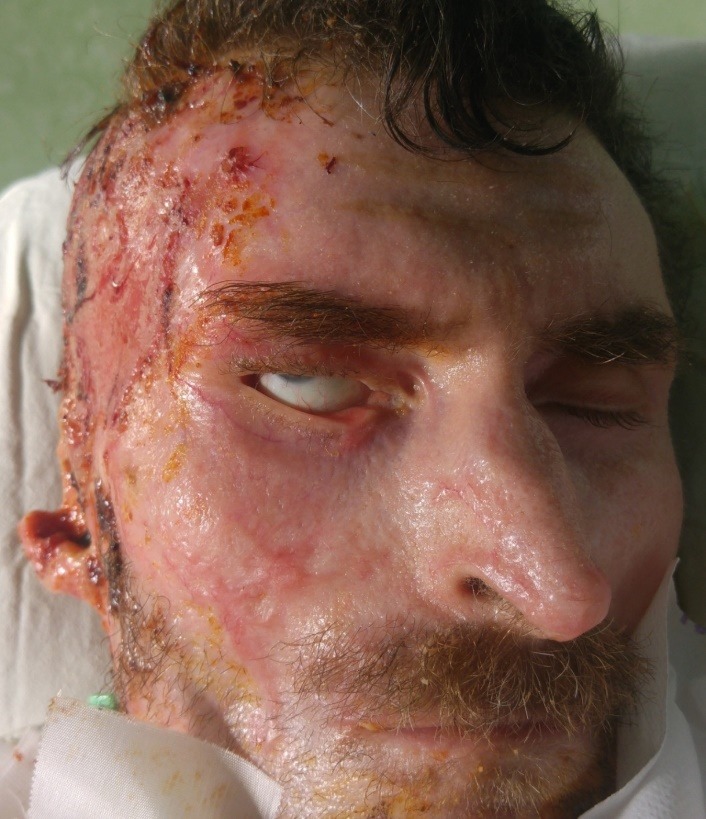
Cicatricial upper and lower eyelid ectropion on the right eye, lagophthalmos with Bell’s phenomenon

**Fig. 13:**
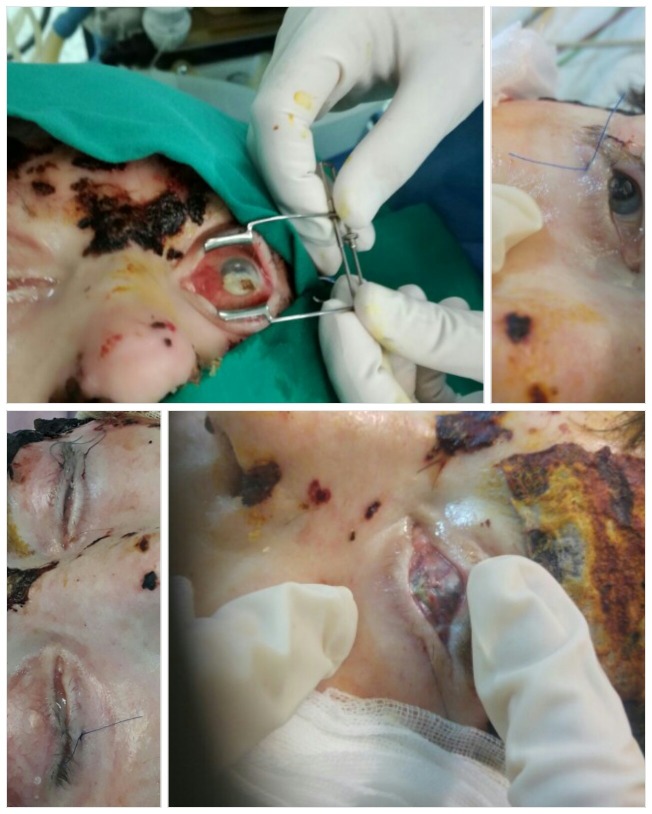
Cicatricial ectropion in the left eye with development of a total corneal abscess, with perforation and iris herniation - a functional rrhaphy; in the right eye, corneal erosion appeared at the inferior corneal limbus, lateral tarsorrhaphy was performed

The rest of three patients developed cicatricial ectropion of various degrees. One 47 years old male patient (**Fig. 11**) developed cicatricial ectropion on the left eye and lagophthalmos in the right eye, on day 65 post-burn injury surgical intervention was performed with excision of the inferior left eyelid scars and skin grafting with full thickness skin graft, with good functional evolution and patient hospital discharge on day 85 post-burn injury. Another male patient, 44 years old (**Fig. 12**) developed cicatricial upper and lower eyelid ectropion on the right eye, lagophthalmos with Bell’s phenomenon on the right eye, he refused the corrective surgical intervention and requested hospital discharge. The last patient of the group is a 59-year-old woman (**Fig. 13**), who after 3 weeks post-injury developed: cicatricial ectropion in the left eye with further development of a total corneal abscess at one month, with perforation and iris herniation - a functional rrhaphy as an edge-to-edge technique was performed on day 31; in the right eye, corneal erosion appeared at the inferior corneal limbus, and a functional rrhaphy was made on day 30 post-injury. Microbiological testing revealed the presence of Pseudomonas Aeruginosa and topical antibiotic treatment was applied according to antibiogram. Ocular evolution was favorable, but the patient developed multisystem organ failure and died on day 51 post-injury. 

## Discussion

The aim of the study was to analyze the spectrum of lesions involving periorbital region, noting the factors that may influence the prognostic of these patients with severe burns in order to improve and reduce the complications and finally decrease the morbidity and mortality and ensure an optimal functional outcome. 

From 210 patients admitted in our burns centre along two years, 82,8% had burns involving the face and 60% had burns involving the face and the periocular region (n=126).

In an Australian study reported by Cabalag et al., from a total of 3340 burns admissions, around 35% suffered facial burns with 12% involving the orbital/ periorbital region [**11**]. In another study on a large number of patients (4758 patients: 2346 adults and 2412 children) from United Kingdom, 18% of the burns involved the face and 2% were periorbital burns requiring surgical treatment [**4**]. As we could observe, our patients had severe injuries, with significant proportion of facial and periorbital involvement, from all our 210 patients 14% also presenting burn ocular injuries and 3% developing secondary ocular complications. This distribution in our patients was generated from a particular infrastructure situation encountered in our country - not having enough specialized centers for burn treatment at national level; the patients with severe lesions being selected and transferred from other parts of the country to our Burn Unit. 

Demographical characteristics (the residence environment, gender, age) of the patient were noted and we could observe a predominance of rural-provenance (55.5%), male gender (66%) and the group of 41-50 years (n=38), but we noticed as well the large group of elderly patients over 61 year 19,5% (n=41). If burn injuries occur in rural areas, a delay was observed in reaching the medical assistance and a prolonged period before admission in the burn unit [**16**].

Male patients are more prone to be involved in traumatic injuries, including burns, similar gender distribution of 2:1 being reported also in other studies [**4**,**17**].

Lundgren et al. analyzed the outcome of elderly patients after burn injuries and observed that the age, as independent parameter (without considering associated comorbidities) along with TBSA and inhalation injuries are the most important factors in the determination of mortality risk post-burn injury during hospitalization and also, presence of patient comorbidities and age over 75 years increase the mortality risk even one year after hospital discharge [**18**].

Extensive burns exceeding 40% TBSA were encountered in 49% of our patients with periorbital involvement, suggesting a large morbidity and mortality rate for this study group, even in highly developed centers, according to international records [**19**].

From our group of 126 patients, 73% presented at least one percent of third degree burn, but a large proportion suffered deep extensive burns - 41 patients (32.5% of the study group) presenting IIIrd degree burns on more than 25% TBSA. Full thickness burns are known as a poor prognosis factor; even more when large surfaces are involved. For full thickness lesions, surgical treatment is mandatory - early excision and grafting in order to avoid serious local and systemic complications and reduce mortality risk [**20**].

A large number of patients had full thickness burns of the periorbital region, 27.8% (n=35), determining a poor recovery prognosis with severe functional impairment. 

In terms of burns etiology, in our group, most of the periorbital lesions had a thermal mechanism, produced by flame and hot liquids in 57 cases and, in 56 cases, involved explosion (usually in our country caused by gas cylinders, as domestic accidents produce in closed space). Explosion injuries are dangerous, determining severe (extensive and deep) burns, involving functional areas with risk of associating inhalation injury and other lesions (craniocerebral trauma, fractures, abdominal trauma), including polytrauma cases, and requesting complex multidisciplinary treatment. Around 4 of 5 patients in our study group presented inhalation injuries.

Blast injuries have a significant risk in producing ocular injuries, with various degrees of severity, trough a primary mechanism of the shockwave or as secondary lesions when exogenous foreign bodies are projected in the eye during the explosion [**21**].

Electric and chemical burn injuries have less frequent occurrence but are associates with severity. In our group of patients with periorbital burns, 7% were electrical and 3% chemical burns; similar data were reported in literature: in the study of Fitzgerald O’Connor et al. the periorbital burns were produced in 4% of the cases by electrocutions and 2% by chemical injuries [**4**].

Besides the lesions determined by electrical flame on the face and orbital region, another pathology appears, such as the formation of cataract within 12 months after the electrocution or less frequently the chorioretinal atrophy [**5**]. Following an electric injury, due to the risk of cataract development, the patient has to be evaluated by the ophthalmologist at the admission, when he is released from the burn unit and at three months interval until the 1 year post-injury. Chemical burns affecting the periorbital region represent serious injuries. From all ophthalmic injuries, literature describes that between 11.5% and 22.1% are chemical burns, produced by alkali or acids [**6**]. Alkali produced more severe injuries than acids because they present lipophilic properties with deep penetration of the tissues, determining saponification, and liquefaction necrosis [**12**].

Adequate immediate treatment is mandatory in periorbital chemical burns: irrigation with large volume of flowing isotonic solutions (more than 30 minutes) in order to remove the substance and interrupt the chemical reaction; the situations that contraindicate the initial irrigation are an observed ocular globe rupture and burn with chemical agents that reacts with lavage solution like calcium oxide (first measure is to perform a dry removal of the substance and then irrigate) [**6**,**12**,**22**]. In our group, there were four male patients with chemical injuries, who presented corneal burns, 3 of them died and one survived. 

Evaluation and specialized ophthalmologic treatment has to start as soon as possible, surgery is needed for the early excision of the deep burns lesions and coverage with autologous skin grafts or skin substitutes.

Analyzing our study group of 126 patients with severe burns involving also the periorbital area, we could observe the presence of multiple negative prognosis factors (elderly patients, extensive burns >40%, full thickness burns affecting the functional areas, unfavorable burn mechanism - electric, chemical injuries or explosions, association of inhalation injuries) leading to severe morbidity and high mortality level (two thirds of those patients died). From those patients, 108 (85.6%) required intubation and mechanical ventilation, with an average of 12.3 days, which worsened the survival rate. A quarter of our patients with injured periorbital area had primary ocular injuries.

As it is known, the eye posses physiologic protective mechanisms represented by blink reflex and Bell’s phenomenon, but those can be overcome in case of aggressive burn injuries with generation of ocular lesions [**12**]. 

Rapid ophthalmologic evaluation after patient admission to Burn Unit is mandatory if periorbital are present in order to start the adequate treatment as quickly as possible. Ophthalmologic medical history was noted, for example in our patient’s case, monophthalmus with past right eye enucleation for post-traumatic ocular complication, another patient presented an atrophic right ocular globe with a band keratopathy from an old lesion, another patient had internal and external pterygium in the right eye and internal pterygium occupying the pupil center in the left eye. 

A critical period for the severely burned patient is represented by the emergency phase -the first 24-72 hours after significant burn injuries - when massive fluid shifts occur, requiring appropriate therapeutic management. Baxter was the promoter of the burn resuscitation using high fluid volumes [**23**-**25**]. 

Burn-shock resuscitation with salt-containing fluids (usually Ringer lactate) is necessary for children or elderly with burns on more than 10% total burn surface area (TBSA) and adult patients with burns on more than 20% TBSA [**26**]. The purpose is to correct postcombustional hypovolemia and hypoperfusion, prevent ischemia, and obtain optimal tissue perfusion in order to avoid further complications [**26**].

Parkland formula (consisting of administration of lactated Ringer’s 4 mL/ kg per % TBSA burn for the first 24 hours, from which half of the volume is administered in the first 8 hours post-burn) is used on wide scale [**27**].

A series of systemic complications can occur as side effects of high volumes fluid resuscitation, including pulmonary edema, acute respiratory distress syndrome, compartment syndrome of abdominal cavity and extremities. Prompt treatment is mandatory in those situations, addressing the correction of systemic complications and surgical decompression interventions for compartment syndrome. Another important complication in patients receiving aggressive burns liquid resuscitation is represented by the risk of developing orbital compartment syndrome (OCS), a serious condition that can lead to complete loss of vision [**28**]. 

Similar to the limbs or abdominal cavity, the orbit is a closed system and orbital compartment syndrome develops when the globe is compressed by the enlargement of the orbital components, without the ability to spontaneous decompression [**5**]. Multiple factors can produce OCS in burn injuries: tissue edema appeared due to extravasations of intravascular fluid and proteins to the extravascular space, corroborated with high volumes of fluids administered in resuscitation protocols and possible presence of inextensible burn eschar [**23**,**28**].

Severe burned patients requiring aggressive fluid resuscitation are usually intubated orotracheal and analgosedated and it is more difficult to perform an adequate clinical examination. Therefore, the patients have to be carefully observed in the acute phase of the burns in order to avoid OCS: in case of burns involving the facial region, the patient’s head has to be elevated with the bed or a pillow and clinical examination has to be performed in a dynamic fashion; if the compartment syndrome develops in unburned limbs or abdomen and associates with decreased blood pressure, a prompt evaluation of the intraocular pressure is mandatory [**5**]. Acute intraocular hypertension can determine anterior ischemic optic neuropathy (AION), further complicated with AION infarction, which, in deep burns may get to bilateral loss of vision [**5**].

Decompression in case of OCS can be obtained performing lateral canthotomy: after dividing the skin, the lateral canthal tendon is sectioned and the lower lid is released from its bony attachment. Increased attention is required due to the distortion of the anatomy encountered in severe burns [**5**].

**Ocular involvement in burns**

It has been demonstrated that patients with periorbital region burns, especially involving the eyelids and eyelashes present a high risk for associating ocular surface injuries and further risk for developing corneal ulceration [**5**].

Ocular globe injuries appear through direct action of thermal or chemical agents on the eye but, according to literature, more often, lesions are produced after corneal exposure due to cicatricial postcombustional retractions [**5**]. 

Both immediate and secondary lesions associated with burns, were also observed in our study, but with a numeric predominance of primary ocular burn lesions (from 37 ocular lesions, 30 were primary and 7 secondary). This 4:1 ratio favoring primary lesions can be explained through high severity burn lesions encountered in our patients with consequent high mortality and impossibility to have a long-term clinical evolution in patients with periorbital injuries. 

**Ocular burn injury primary involvement**

We had 30 patients with corneal injuries, representing almost a quarter of periorbital burn injuries and 14.3% of all the patients admitted in two years in our burn unit. We observed an even higher mortality rate in this subgroup (83%). The patients had different Ropper-Hall grades and all the 12 patients with corneal injuries grade II-IV died, also having severe burn injuries.

In this subgroup, the predominance was for male gender (three quarters of them), 9 patients (30%) had more than 60 years old, 20 patients (66%) had lesions exceeding 40% TBSA. Ocular burns (n=30) etiology was explosion in 11 patients, 4 chemical burns, 2 electric injuries, and the rest of 13 were thermal injuries. Five patients, all males, had work related accidents, 3 being chemical burns and the other 2 electric burns. Inhalation injury was encountered in 25 of those patients; 10 patients associated explosion mechanism and inhalation injury. Electric burns produced a Roper-Hall grade 1 injury in one patient and 2 in the other patient. In cases of chemical burns, from the three sulfuric acid burns, one patient, who survived, had a Roper Hall grade 1 corneal injury with a good clinical evolution with topical treatment and the other two (work related accidents), both dead, presented Ropper Hall grade 3 injuries. The patient with extensive caustic soda burn injuries (work related accident) on 60% TBSA also associated severe corneal burns, a Roper Hall grade 4; he died on day 8 post-injury.

As we could observe, this subgroup showed severity characteristics that led to a very poor prognosis, attested by the high-level mortality rate. The average period of hospitalization was 20 days (a median of 14.5 days), half of the patients died in first 2 weeks following injuries; 27 patients (90%) required intubation and mechanical ventilation, with an average of 18 days. 

In the particular situation illustrated by this subgroup, of ocular lesions occurrence in the context of severe burnt patients with a highly reserved vital prognosis, it is difficult to draw a conclusion on the long term evolution of the cases, due to the low number of survivals (5 patients), all of them having Roper Hall grade 1 corneal lesion, with favorable long term outcome. Also, the association of systemic postcombustional complication and extensive TBSA involvement made the adequate surgical treatment of early excision and skin grafting of deep lesions a challenge in approach in some cases. A proof in this direction is the small number of surgical interventions (2 cases) addressing the periorbital region in this subgroup. In one of those patients, with Roper Hall grade 3 corneal burns, after full face skin grafting, including palpebral region, in evolution, beside systemic severe complications, lagophthalmos marked by scarring ectropion of upper and lower eyelids developed, tars had deficient structure and could not be rafted, with rigid conjunctiva in the lower part, with bilateral exposure keratitis and corneal ulcer complication in the right eye. The patient died before a specialized reconstructive treatment could be applied. 

In the patients with ocular involvement, in our Burn Unit, an eye examination is performed with regularity, topical treatment prescribed by the ophthalmologist is applied including artificial tears, anti-inflammatory agents if required, antibiotic topical agents (according to antibiograms), and topical anesthetics are sometimes needed for pain reduction. Microbiological testing is performed, such as screening on admission, then once a week or if suggestive clinical signs occur and are specific to an infectious pathology.

**Secondary ocular complications**

In our study, secondary ocular complications developed in seven cases during their long-term hospitalization and mechanical ventilation (an average of 72 hospitalization days): cicatricial ectropion with various degrees of severity, lagophthalmos with exposure keratitis, keratopathies with one inferior corneal ulcer with superficial corneal abscess and one total corneal abscess, with perforation and iris herniation. As surgical treatment, blepharorrhaphies were performed in 2 patients and one full thickness skin graft to the lower eyelid to provide adequate globe coverage.

Exposure keratopathy determining epithelial defects precedes the formation of epithelial ulcerations (ulcerative keratopathy). Daily thorough examinations of epithelial defects are required in burn patients, as well as the correction of determinant causes. Examination under magnification is mandatory, and if a stromal opacification appears, there is certitude of corneal ulcer, which is important to be correctly treated to avoid the vision loss. Descemet membrane lies in the posterior corneal stroma, and is a strong structure that, in near full thickness corneal lesions may hold the aqueous humor and create the aspect of descemetocele. This is a very severe situation, preceding the corneal perforation [**5**]. In complicated cases, enucleation may be needed [**12**]. 

An important risk factor in developing exposure keratopathy, besides the presence of periocular lesions determining eyelid retraction, is the need for intubation and mechanical ventilation. Bird and co. reported the occurrence of exposure keratopathy in 37–57% of the patients hospitalized in intensive care units, requiring sedation or intubation [**29**].

Multiple causes lead to exposure keratopathy in critical patients, including burns victims: sedation, reduced consciousness (as sedation effect or in neurologic injuries), mechanical ventilation, miorelaxing agents, incomplete eye closure, reduced blink and tear production, affected corneal reflex and increased vascular permeability [**30**].

Those risk factors were also encountered in our patients, with all seven patients with secondary ocular complications requiring long-term intubation and mechanical ventilation (with an average of around four weeks). 

In burned patients, the risk of infection of a corneal ulcer may occur, requiring adequate microbiological testing and treatment according to antibiograms. In bacterial keratitis, the germ pathogenicity plays an important role in the prognosis of these infections, and several aggressive, multiresistant bacteria can be selected during long-term hospitalization, infections with Acinetobacter, Staphylococcus and Pseudomonas being reported by various studies [**5**]. The same infectious agent was also encountered in our patients. Bacterial keratitis has a fulminant course with a worse prognosis than sterile corneal ulcers [**5**].

Also, keratitis with other infectious agents such as fungal organisms or viruses may occur in burn patients due to the impairment of their immune system as a response to severe burn injuries. It is important to correctly diagnose and treat a fungal keratitis (usually involving Candida) to avoid further ocular complications. Due to long-term immunosuppression, herpetic keratitis may also be a problem in severely burned patients [**5**].

Definitely, the most important thing in severely burned patients requiring long-term hospitalization is to apply preventive measures to avoid ocular complications; also, through regulate ophthalmologic evaluations if an exposure keratitis is detected, prompt treatment being mandatory to avoid functional impairment including loss of vision. 

## Conclusions

The involvement of face in major burns occurs frequently with important functional and aesthetic negative consequences. Severe burns of the periorbital region, usually associated with other facial lesions can be devastating for the patient’s clinical evolution. The severity of the periorbital burn injury and future prognostic depends on patient characteristics, mechanism of injury, duration of exposure to traumatic agent, burn extension and depth, associated lesions, infectious risk, and the quality of the treatment applied.

Presence of ocular injuries in various severity degrees imposes an adequate evaluation and specialized treatment, being associated with important morbidity. Severely burned patients need prolonged hospitalization; therefore, it is mandatory to apply preventive measures to avoid ocular complications. If exposure keratopathy is detected, prompt ophthalmologic treatment is essential to avoid functional impairment including loss of vision.

The final purpose in therapeutic management of the periorbital burns is the functional and aesthetic recovery with the primary objective being the maintenance of the vision in order to restore an optimal quality of life and chance of social reintegration for the burn victim. 

**Disclosures**

None. 
